# Air Pollution and Workplace Choice: Evidence from China

**DOI:** 10.3390/ijerph19148732

**Published:** 2022-07-18

**Authors:** Tao Lin, Wenhao Qian, Hongwei Wang, Yu Feng

**Affiliations:** 1 School of Economics, Shanghai University of Finance and Economics, 777 Guoding Rd., Shanghai 200433, China; lintao@163.sufe.edu.cn; 2 School of Public Economics and Administration, Shanghai University of Finance and Economics, 777 Guoding Rd., Shanghai 200433, China; wangjue@mail.shufe.edu.cn (H.W.); fengyu101296@outlook.com (Y.F.)

**Keywords:** air pollution, workplace, avoidance behavior, public health, regression discontinuity design, China

## Abstract

Understanding the impacts of air pollution on public health and individual behavior is crucial for optimal environmental policy design. Using 2015 census microdata in China, this paper examined the causal effect of air pollution on working place choice. The research design relies on a regression discontinuity design based on China’s Huai River Policy. The discontinuity in air pollution caused by the Huai River Policy provides a natural experiment to estimate the impact of air pollution. The results show that air pollution significantly increases the possibility of individuals working near home. The positive effect of air pollution on working near home is more significant for women, the elderly, urban individuals and those individuals working in secondary and tertiary industries. This study improves our understanding of the health effects and avoidance behavior associated with environmental hazards, discusses the negative impact of air pollution on labor mobility and mismatch by making individuals work nearby, and emphasizes that strengthening air pollution control should be a long-term policy.

## 1. Introduction

Air pollution is considered as a major issue for the community in China. In recent years, the problem of air pollution caused by China’s rapid, extensive, and low-quality economic development has attracted great attention from the government and society. According to the 2019 China Ecological Environment Status Bulletin, a total of 218 days of severe and serious pollution have occurred in 337 prefecture-level and above cities across the country, of which the number of days of haze with PM_2.5_ as the primary pollutant accounted for 78.8%. In 2019, the World Health Organization (WHO) has announced ten major threats to human health, among which air pollution ranks first. Epidemiological studies show that inhaling polluted air will lead to pathological changes in the lung and respiratory system, chronic damage to organs such as the heart and brain, resulting in cancer, stroke, heart and brain diseases [1].

Theoretically, there has been a growing body of economic literature on the impact of air pollution on human capital, including physical health [2,3,4], mental health [5,6], cognitive performance [7,8], and productivity [9,10,11]. Chen et al. [2] and Ebenstein et al. [12] provided the first quasi-experimental evidence of the impacts of sustained exposure to air pollution focused on China. They examined the impact of sustained pollution exposure on life expectancy, based on China’s central winter heating system, and found that the winter heating policy raised PM_10_ levels by 46 percent in the region north of the Huai River between 2004 and 2012, causing a reduction in life expectancy of 3.4 years. Chang et al. [11] studied workers in the service sector, where jobs may be more cognitively demanding than those in the manufacturing sector. Using daily performance data for workers in two call centers in China, the authors estimated that a 10-unit increase in the Air Pollution Index (API) decreases the number of daily calls handled by a worker by 0.35 percent. These studies have reached the consensus that there exist negative effects of air pollution on human capital and productivity.

Facing high levels of pollution, individuals can take preventive measures to reduce exposure and mitigate the impact, such as defensive spending (e.g., face masks and air purifiers) [13,14] and migration [15,16]. Using sales indices for face masks and air purifiers from China’s largest ecommerce platform, Taobao, Sun et al. [13] showed that people buy more face masks and air purifiers when ambient pollution levels exceed key alert thresholds. However, risk-compensating and avoidance behaviors in mitigation and adaptation are not adequately considered due to data limitations and identification problems. Increasing defense spending is not the only, nor is it a major preventive measure [17]. Due to the household registration system and high housing prices, it is difficult to migrate to cleaner cities in China [17]. Therefore, it is of theoretical and practical significance to explore how to avoid the impact of air pollution when air pollution is difficult to be controlled in the short term.

On the other hand, workplace choice is not only an important individual behavior, but also an important economic concept in labor economics. Choosing where to live and work will affect the welfare of individuals [18,19]. Whether the labor force can freely choose the place of work not only affects labor mobility, but also has an important impact on economic development [20]. Existing studies have studied the determinants of workplace choice from various aspects, such as income, education, and preference [21,22,23]. Using data of 833 knowledge-workers in high-technology and financial services, Frenkel et al. [23] investigated the residential location and workplace choice of knowledge-workers at the intra-metropolitan level by applying discrete choice models. They found that the most important factors are municipal socioeconomic level, housing affordability, and commuting time, while substantial secondary factors are cultural and educational land-use and culture-oriented lifestyle. However, there is little literature on the relationship between environment and workplace choice. Especially, when the environmental quality is deteriorating, workplace selection may become a way to avoid pollution risks. To reduce exposure, does air pollution affect people’s workplace choice? We provide an answer to this question in the context of China.

This paper examined the causal effect of air pollution on working place choice. The data link the 2015 census microdata at the individual level with air pollution at the county level. We estimated the effect of air pollution on working near home using a regression discontinuity design (RD) based on China’s Huai River Policy [2,12,14]. The policy dictates that areas to the north of the Huai River receive free or highly subsidized coal for indoor heating. This has led to the construction of a coal-powered centralized heating infrastructure only in cities north of the Huai River, with no equivalent system in cities to the south. The central heating system generates considerable air pollutants during coal combustion. The Huai River Policy provides a compelling natural experiment to estimate the causal effects of air pollution on working near home. 

We obtained several findings. First, there is strong evidence that the air quality is deteriorating north of the Huai River. On average, the Huai River Policy increases PM_10_ concentrations in the north by 13.2 percent. Second, we found that the Huai River Policy has a large and statistically significant positive impact on working near home. The Huai River Policy increases the probability of working near home in the north by 5.6 percent on average. Third, we found that an additional 10 μg/m^3^ of PM_10_ significantly increases the probability of working near home by 13.6 percent. Fourth, the positive effect of air pollution on working near home is more significant for women, the elderly, urban respondents, and those individuals who work in secondary industries. These findings are consistent with the existing literature that higher air pollution is associated with poorer health, higher mortality, and better self-protection [5,6,14]. 

We make two contributions to the literature. First, we contribute to a new but growing body of literature that identifies the coping strategies for environmental shocks. Relevant work in this body of literature has detected the role of risk-compensating behaviors, such as pollution information, household location choices, avoidance actions, and defensive spending (e.g., face masks and air purifiers), in helping households and individuals to cope with environmental shocks and reduce pollution exposure [13,14,24]. To our knowledge, our paper is the first to provide evidence on how changes in pollution levels affect work site selection. Individuals facing high levels of pollution may choose to work near home to reduce exposure and mitigate the impact. Although some empirical evidence confirms that households choose locations to seek a better environmental quality (i.e., sorting, migration) [15,25,26,27,28,29,30,31], migration is a long-term choice of a family and limited by many socio-economic factors (e.g., housing prices and health care). We argue that working near home may be a more common choice to avoid pollution than migration, especially due to China’s generally high house prices and hukou restrictions. Our study extends the literature on pollution avoidance behavior.

Second, we contribute to the literature on labor mobility. This directly connects to the existing studies on the factors that drive labor mobility and cause labor spatial mismatch. Many scholars have studied this problem from many aspects (e.g., human capital and migration cost) [32,33,34,35]. We try to answer this question from a new perspective. We argue that outdoor air pollution reduces the cross-city mobility of labor and the possibility of cross-regional work. Our empirical results also verify this. In areas with more serious pollution, the labor force tends to work in local cities rather than across regions, which means that in areas with more serious air pollution, there is lower labor mobility. This may be an important reason for labor spatial mismatch [36,37] and market segmentation [38,39], as well as further widening of the income gap and unbalanced development among regions [20,40].

The rest of this paper is organized as follows. Section 2 provides background on the Huai River Policy and its recent reform. Section 3 introduces the data sources and variable design. Section 4 introduces the empirical strategy. Section 5 discusses the causal impact of air pollution on working near home. Section 6 presents the heterogeneity analysis. Section 7 discusses our results. Section 8 concludes this paper.

## 2. Institutional Background: Huai River Policy

As northern China is very cold in winter, respondents use various forms of heating. The traditional heating method in China is to burn loose coal in a stove. China’s central heating system began in the 1950s. Referring to the heating mode of the Soviet Union, China initially established a central heating system mainly using coal as fuel.

Due to resource and budget constraints, central heating gives priority to the cold northern region, which is limited to cities in north, northeast and northwest China. Specifically, the Qinling Mountains and Huai River are the dividing line (the average temperature of this line in January is about 0 °C). Cities north of the Huai River have central heating, while cities south of the Huai River do not have heating. This is also known as the Huai River Policy.

Before 1978, subject to the level of economic development, the development speed of urban central heating was quite slow. Since the reform and opening up in 1978, China has gradually transitioned from a planned economy to a market economy. Many private sectors began to be born on a large scale, and the central heating system also entered a period of great development. By 2003, most northern cities in China had built central heating systems.

With the rapid growth of the urban central heating area, the financial burden of northern cities is increasing. The commercialization reform of heating implemented by the government in 2003 also changed the original free heating system [41]. The government abolished the welfare policy of free heating and began to charge for heating. In terms of charge management, the government still provides heating subsidies for employees of state-owned enterprises and institutions, but employees of non-state-owned enterprises do not enjoy the benefits. The commercialization of heating increases the heat cost of respondents. However, with the growth of urban construction and personal income, China’s urban central heating area still maintains a stable and rapid growth. Due to the reform of the heating policy, coal consumption in northern China continues to grow [12,14]. Since 2003, although the central heating system has increased the household heating cost, it has not significantly reduced the household heating demand. Central heating in the northern regions in winter is still dominated by coal-fired heating.

**Figure 1:**
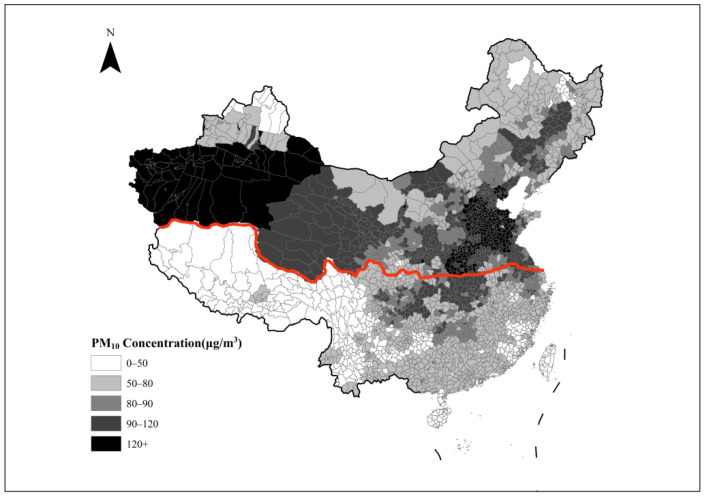
Huai River Policy line and PM_10_ concentrations. Note: The red line is China’s Huai River/Qinling Mountain Range winter heating policy line. The polygons represent counties, the third level of the administrative hierarchy in China. The coloring of the polygons corresponds to average PM_10_ concentrations from 2014 to 2015.

However, with the rapid growth of the urban heating area, the heating mode with coal as the main fuel means that the air pollutants (soot, sulfur dioxide, etc.) produced by coal combustion also increase synchronously. Based on the quasi-natural experiment of the central heating policy in northern China, some scholars have found, through an RD design, that central heating leads to more serious air pollution in northern China [2,12,14,42]. This seems to be a contradiction and trade-off between heating and air quality. Figure 1 shows the locations of the Huai River (red line) and the air quality. It is clear that counties in northern China are much more polluted than those in southern China.

In order to protect the environment and reduce the impact of pollution on respondents’ health, the government has implemented many projects and policies to reduce the emission of pollutants. One of the biggest is the replacement of coal with natural gas or electricity as primary fuels for heating [43]. In 2013, Beijing first proposed and implemented the coal-to-gas policy, replacing coal with natural gas or electricity for central heating in urban areas, and providing subsidies to encourage families to replace coal-fired heating in rural areas. Later, other regions such as Tianjin and Hebei also launched the coal-to-gas policy in 2015 and 2016. It should be noted that despite the policy of changing coal to gas, the northern region still mainly depends on coal combustion to realize central heating in winter [43].

## 3. Data

### 3.1. Population Sample Survey Data

We mainly used the sampling survey data of 1% of China’s population in 2015. These are nationwide data. China’s National Bureau of Statistics conducted a national 1% population sampling survey at 0:00 on 1 November 2015. The survey takes the whole country as the whole and prefecture-level cities (regions, leagues, and prefectures) as the subpopulation. The stratification, two-stage, probability proportion and cluster sampling methods were adopted. The final sample size was 21.31 million, accounting for 1.55% of the total population of the country. The census takes the individual as the unit, and counts a number of indicators (e.g., gender and education). Additionally, it also counts a number of indicators of the family characteristics (e.g., hukou and family registration). The individual work information and characteristic data used in this paper are from this population sample survey.

### 3.2. Air Pollution

We collected the historical data of 1482 air monitoring stations in China from the air quality online monitoring and analysis platform (The real-time data are published on the following website: http://www.aqistudy.cn/ (accessed on 1 May 2021)). The platform is the largest real-time air quality monitoring network ever built in China, implementing the full coverage of municipalities, provincial capitals, cities with independent planning, all prefecture-level cities, key environmental protection cities, and environmental protection model cities. The data include the PM_10_ concentration per hour from 1 November 2014 to 30 October 2015. We calculated the pollution data of each county according to the monitoring information of each monitoring station. First, we averaged the hourly data of each monitoring station to obtain the daily monitoring results of each monitoring station. Then, taking the county as the center, we calculated the weighted average of air quality variable at each air monitoring station within 100 km to obtain the pollution index of each county every day. Among them, the reciprocal of the distance from each monitoring station to the county center was taken as the weight. Finally, we calculated the mean value of air quality indicators in each county during the sample period and finally obtained the PM_10_ concentration of 2501 counties in China.

### 3.3. Heating City

The list of heating cities is mainly from the statistical yearbook of China’s urban construction. In order to be consistent with the statistical range of household mortality, we mainly used the list of heating cities in 2014. Among 295 prefecture-level and above cities in China, 130 northern cities had central heating, and the other 165 southern cities had no central heating system. Although the list of heating cities will change every year, the change is very small, only increasing or decreasing by one or two cities. Overall, the list of urban heating in China remains basically unchanged.

### 3.4. Control Variable

We controlled some individual characteristics, including gender, age, marriage, ethnicity, and type of hukou. We also controlled the meteorological conditions. The meteorological data come from the China meteorological data network (see http://data.cma.cn (accessed on 10 May 2021)). The original data are the observation data of 840 meteorological stations in China, including rainfall, average wind speed, air pressure, and minimum and maximum temperatures. We mainly used temperature, precipitation, relative humidity, and wind speed for control since they are the main meteorological factors affecting air pollutants. In order to calculate the above four variables of each county, we first interpolated the meteorological station data according to the inverse distance weighting (IDW) method to obtain a 1km×1km grid layer across the country. Then, we extracted the above four variables of each county center based on this layer. Finally, we obtained the average temperature, precipitation, relative humidity, and wind speed of each county. The descriptive statistics of the variables are shown in Appendix A Table A1.

## 4. Empirical Strategy

Formally, a linear regression equation for the impact of air pollution on working near home was estimated as shown below:

(1) $$Work_{ci}=\beta_{0}+\beta_{1}Pollution_{c}+\varphi X_{ci}+\varepsilon_{ci}$$

where subscripts *c* and *i* represent counties and respondents, respectively; the dependent variable *Work* is an indicator variable that equals one if respondent *i* works and lives on the same street, and zero otherwise; *Pollution* is the PM_10_ concentration of county *c*; *X* is the vector of observable features that may affect working place selection; ε is the disturbance term; and the coefficient *β*_1_ measures the effect of PM_10_ exposure on working place selection after controlling for the available covariates. 

The key challenge in estimating the causal impact of air pollution on working place choice is that variations in air pollution could be endogenous. Consistent estimation of *β*_1_ requires that the unobserved determinants of working place selection do not covary with *Pollution* after adjustment for the observed covariates, but the validity of this assumption has been questioned by previous research. For example, air pollution levels are often associated with complex meteorological processes that can directly affect human health, and it is difficult to control for all these factors [12,17]. Other unobserved socio-economic factors (e.g., income) could also confound the impact of air pollution on working near home. Furthermore, pollution concentrations are prone to measurement error, which will attenuate the coefficient associated with PM_10_. Therefore, the OLS estimate of *β*_1_ is likely to be biased.

We addressed the potential endogeneity issue by constructing a regression discontinuity (RD) design based on the Huai River Policy (akin to existing studies such as Chen et al. [2], Ito and Zhang [14], and Ebenstein et al. [12]). As shown in Section 2, this policy provides free or heavily subsidized coal for heating north of the river but no subsidies to the south. This has led to the construction of a coal-powered centralized heating infrastructure only in cities north of the Huai River, with no equivalent system in cities to the south. Therefore, northern cities face more serious air pollution [12]. Near the Huai River boundary, the counties in the south become the opposite of the counties in the north. By comparing the difference in local air pollution caused by the Huai River Policy, we can estimate the local average treatment effect (LATE) of air pollution on individual workplace selection [2]. RD design is a quasi-experimental research design which could address the previous literature’s limitations and provide a clear identification. Following Chen et al. [2] and Ebenstein et al. [12], we examined whether the Huai River Policy causes discontinuous changes in PM_10_ concentrations and the probability of working near home north of the river using the following specifications:

(2) $$Pollution_{c}=\alpha_{0}+\alpha_{1}D_{c}+\alpha_{2}f(Dist_{c})+\kappa X_{ci}+\nu_{ci}$$

(3) $$Work_{ci}=\delta_{0}+\delta_{1}D_{c}+\delta_{2}f(Dist_{c})+\gamma X_{ci}+\mu_{ci}$$

where *Dist* is the running variable, representing the shortest distance (in km) from each county to the Huai River, taking positive values for counties to the north of the Huai River and negative values for counties to the south; *D* is an indicator variable equal to one for counties with a positive value of *Dist*; *f*(*Dist*) is a local regression function in *Dist* that allows the relationship between outcomes and the running variable (*Dist*) to vary on either side of the cutoff; in all our specifications, we also controlled for a vector of covariates (*X*), including demographic variables and meteorological conditions such as gender, ethnicity, age, marriage, hukou type, temperature, precipitation, relative humidity, and wind speed; *μ* and *ν* are the error terms.

The parameters of interest are *α*_1_ and *δ*_1_, which provide an estimate of whether there exist discontinuities in PM_10_ and the probability of working near home north of the river, after flexible adjustment for the covariates. If the key assumptions of the RD are satisfied, the estimated *α*_1_ and *δ*_1_ reveal the causal effect of the Huai River Policy on *Pollution* and *Work*. 

The parameters *α*_1_ and *δ*_1_ can be identified by both non-parametric and parametric methods. In this paper, we emphasize the results using the non-parametric approach, as the parametric RD approach is found to have several undesirable statistical properties [43,44]. In practice, the key of the RD design is to select the optimal bandwidth to localize the regression fit near the cutoff. The choice of bandwidth involves balancing the conflicting goals of focusing comparisons close to the cutoff (for the “bias” concern) and having a large enough sample for reliable estimation (for the “precision” concern). We used the mean squared error (MSE) optimal and data-driven bandwidth selection methods (following Calonico et al. [45]; Calonico et al. [46]) and different kernel functions (i.e., triangular, epanech., and uniform) to calculate the optimal bandwidth. For all RD estimations, we estimated local linear regressions using observations within an optimal bandwidth. All standard errors were clustered at the county level. 

There are two key assumptions for RD designs. One is that the treatment status is determined by a random assignment or forcing variable and cannot be manipulated [47]. In our design, the forcing variable is the shortest distance (in km) from each county to the Huai River, which cannot be manipulated, but we still give some evidence. Appendix A Figure A6 shows the histogram of county distance with a kernel density estimate, and Appendix A Figure A7 shows the McCrary test [48] (the McCrary test is an important test used to check whether there is any jump in the density of the forcing variable). We found that the density of *Dist* moves smoothly around the threshold. The second assumption is that any unobserved determinants of PM_10_ or whether respondents work near home may change smoothly as they cross the Huai River. In the Result section, we show that a variety of work- and pollution-related local characteristics (i.e., covariates) (we include two main sets of covariates that might be related to the outcome variables; the first set is a vector of weather variables, and the second set is a vector of the demographic characteristics) are smooth functions across the threshold. Additionally, we used non-parametric RD estimation involving additional covariates to increase the efficiency of the estimator (if the relevant assumption is not fully satisfied, adjustment for control variables could remove potential sources of bias and allow for causal inference. In addition, including balanced covariates in RD estimation could also increase the precision of the RD estimator) [49]. 

Next, we used a fuzzy RD approach [46,49,50] to estimate the impact of air pollution on working near home. This approach is used to assess the impact of an imperfect binary treatment where the probability of treatment rises at some threshold, but being above or below the threshold does not fully determine treatment status (i.e., imperfect compliance). In our context, exposure to PM_10_ increases significantly to the north of the Huai River, but pollution exists both north and south of the river, making our context naturally analogous to a fuzzy RD [51]. 

The fuzzy RD estimates can be estimated by taking the ratio of the estimated discontinuity in the probability of working near home to the estimated discontinuity in PM_10_, by local linear regression at the Huai River (see Calonico et al. [51]). Actually, this result is an instrumental variable method, in which PM_10_ is instrumented by the Huai River Policy. The fuzzy RD estimates of the impact of PM_10_ on working near home are analogous to the 2SLS estimates [46,49,50]. Specifically, if the Huai River Policy only influences respondents working near home through its impact on PM_10_, an important appeal of the results is that they produce estimates of the impact of units of PM_10_, so the results are applicable in other settings (e.g., other developing countries with comparable impacts of units of PM_10_ concentrations). 

## 5. Results 

### 5.1. Summary Statistics and Graphical Analysis

Table 1 presents the summary statistics for the main variables and provides evidence on the validity of the RD design. Columns (1) and (2) report the mean values and SDs to the north and south of the Huai River. Column (3) documents the mean difference between the north and the south along with the standard error, and column (4) reports the discontinuous changes and standard errors along the Huai River using local linear regression. 

**Figure 2:**
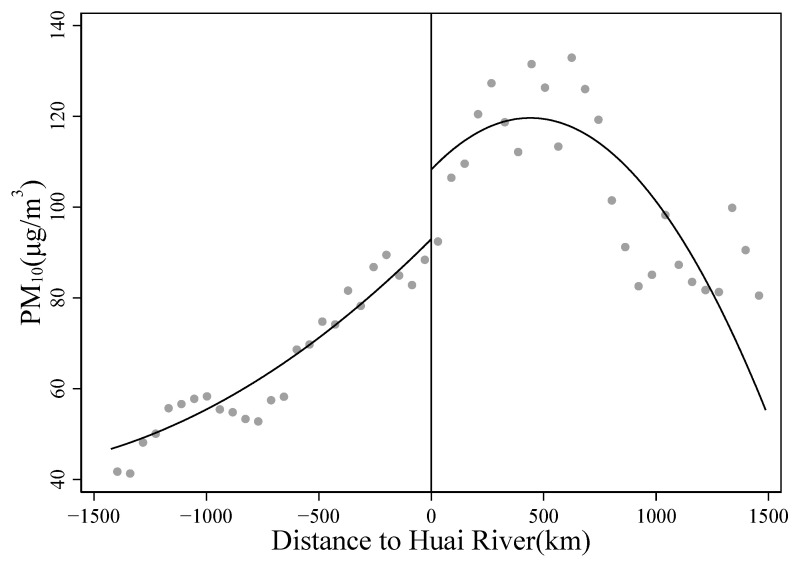
RD plot for PM_10_. Note: The figure plots the binned averages of county-level PM_10_ concentrations against the running variable. The solid line represents a quartic polynomial fit of PM_10_ on each side of the threshold.

We begin the analyses with an assessment of the Huai River Policy’s impact on PM_10_. Panel A shows large differences in PM_10_ concentrations between the south and the north of the Huai River. According to the local linear RD estimates, the north-south difference in PM_10_ along the Huai River is 11.2 μg/m^3^. In Figure 2, we plot the binned averages of county-level PM_10_ concentrations against the distance from the county centroid to the Huai River. We also plot the polynomial fit of PM_10_ against the running variable. It is clear that PM_10_ has a discontinuous jump to the north of the river. 

**Figure 3:**
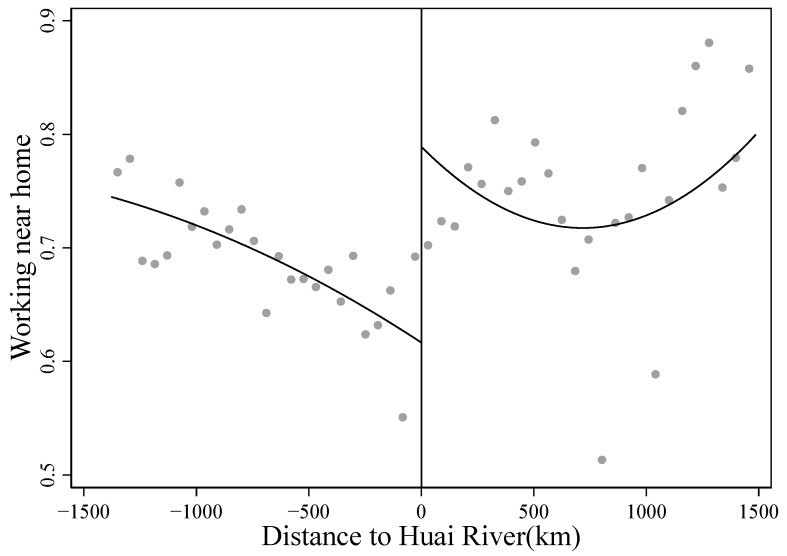
RD plot for work. Note: The figure shows the binned averages of working near home against the running variable. The solid line represents a quartic polynomial fit of working near home on each side of the threshold.

Similarly, we observed a large decline in the probability of working near home along the Huai River. Column (3) in Panel B of Table 1 shows that the share of respondents working near home in the north is much higher than in the south by 0.112. Column (4) shows that the local linear adjusted differences are 5.3 percent. In Figure 3, we plot the binned averages of working near home against the distance from the county centroid to the Huai River and its polynomial fit. An upward jump in the share of working near home is observed to the north of the river. 

Additionally, though the RD design’s identification assumption that unobservables change smoothly at the boundary is impossible to be tested directly, it would be reassuring if observable determinants change smoothly at the boundary. We tested a rich set of demographic characteristics and weather variables and present them in Panels C and D of Table 1. We found that, though there are differences between the south and north of the Huai River, the differences from the local linear regressions are much smaller and statistically insignificant at the boundary. 

### 5.2. Impact of the Huai River Policy

Table 2 presents the RD estimates of PM_10_ and working near home along the Huai River using local linear regression. We used the mean squared error optimal bandwidth method (MSE) proposed by Calonico et al. [49] and Calonico et al. [46]. Each RD estimate also has the optimal bandwidth for both sides of the threshold and all standard errors are clustered at the county level. Columns (1)–(3) report the RD results using the three different kernel functions without inclusion of any other control variables. In Columns (4)–(6), we present the results for the same three regressions but with demographic and weather controls.

Different kernel functions and the inclusion of control variables did not significantly change the estimate. We emphasize the estimates from the most comprehensive specification in Column (4). Panel A of Table 2 shows that the impact of the Huai River Policy on PM_10_. We found that the Huai River Policy increases PM_10_ concentrations in the north by 11.8 μg/m^3^ on average, which is equivalent to an increase of 13.2 percent in the mean in the regression sample (given the average PM_10_ is 89.2 μg/m^3^). 

Panel B of Table 2 reports the RD estimates for working near home. We found that the Huai River Policy has a large and statistically significant positive impact on working near home. Specifically, the Huai River Policy increases the probability of working near home in the north by 5.6 percent on average, which is equivalent to an increase of 8 percent (given the sample mean is 0.702). These regression results echo the graphical results that the Huai River Policy causes a significant deterioration in the air quality and an increase in the probability of working near home in northern China.

### 5.3. Impact of PM_10_ on Work

Table 3 presents the estimated effects of an additional 10-μg/m^3^ increase in PM_10_ exposure on respondents working near home. Panel A reports the fuzzy RD estimates using three different kernel functions. To make sure that our analyses are not sensitive to different specifications, we estimated the fuzzy RD results without and with weather and demographic characteristics. We found that the RD results are reasonably robust for different kernel functions and control variables. We present the results in Column (4), where the triangular kernel function is used and both demographic and weather conditions are controlled. Panel A shows that an additional 10 μg/m^3^ in PM_10_ significantly increases the probability of working near home by 13.6 percent. This observation is consistent with the results in the previous section and indicates that the Huai River Policy affects the probability of respondents working near home via its impact on PM_10_. 

Panel B reports the OLS results for comparison. The estimate in Column (2) of Panel B implies that a 10-point increase in PM_10_ is associated with a 0.5% increase in the probability of working near home. In addition, it is worth emphasizing that, relative to the OLS estimates, the fuzzy RD estimates are more stable and larger in magnitudes, suggesting that OLS estimates are biased downward possibly due primarily to omitted variables and/or measurement errors. These findings are remarkably stable and are not affected by the inclusion of different controls and alternative ways to estimate the RD coefficient and standard errors. 

### 5.4. Robustness Checks

We conducted several robustness checks to help assess the validity of our results. First, we used the air quality index (AQI) as an independent variable and re-estimate. We used the PM_10_ concentration in the main tables because PM_10_ is the main pollutant produced by coal-fired heating. Coal combustion produces a variety of pollutants. We used the overall measure of ambient air quality, the AQI, to measure pollution. Six air pollutants (i.e., PM_2.5_, PM_10_, SO_2_, NO_2_, CO, and O_3_) were used to compute the AQI (the Ministry of Environmental Protection: http://www.mee.gov.cn/ (accessed on 10 May 2021)). In reality, the Ministry of Environmental Protection (MEP) often uses it to inform the public of pollution levels [43]. The larger the AQI score, the higher the air pollution level. Appendix A Table A2 reports the results using the AQI. In general, we found that the results are similar in sign and magnitude to those in Table 2 and Table 3. 

**Figure A1:**
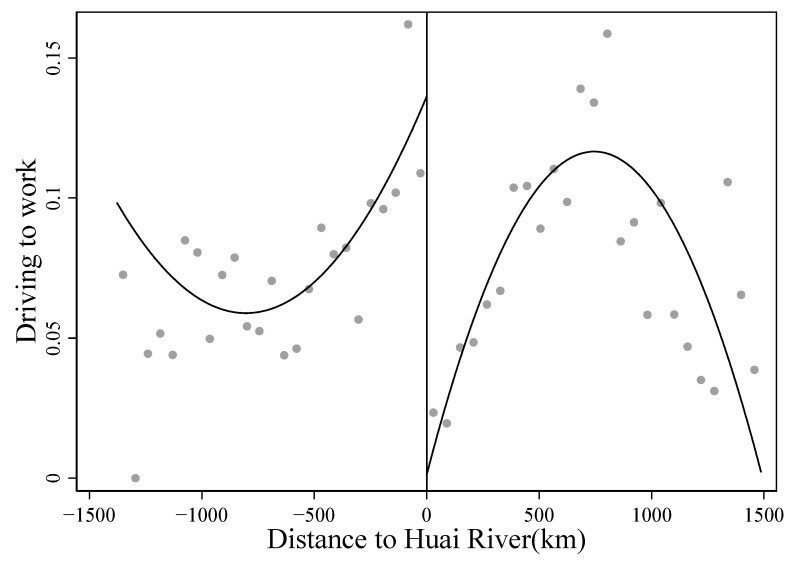
RD plot for driving to work. Notes: The figure shows the binned averages of driving to work against the running variable. The solid line represents a quartic polynomial fit of driving to work by car on each side of the threshold.

**Figure A2:**
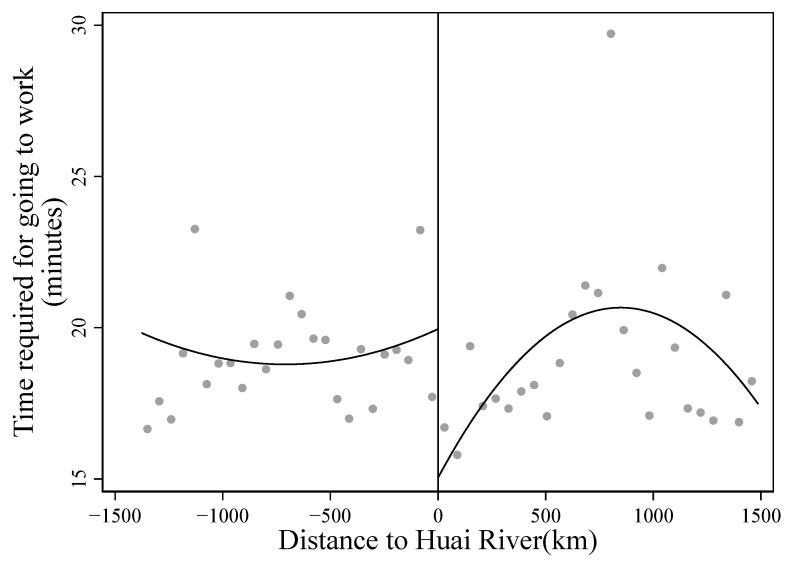
RD plot for the time required for going to work. Notes: The figure shows the binned averages of the time required for going to work against the running variable. The solid line represents a quartic polynomial fit of time on each side of the threshold.

Second, we constructed two alternative measures for respondents working near home: whether respondents drive to work (Yes = 1) and the time required for going to work (in minutes). If one works near home, this means that they are less likely to drive to work, and that the time required to go to work should be shorter. Appendix A Figure A1 and Figure A2 present the RD plots. Table A3 reports the estimated results. We found air pollution significantly decreases the probability of respondent driving to work and the time required for going to work. These results support our findings in the main analysis.

**Figure A3:**
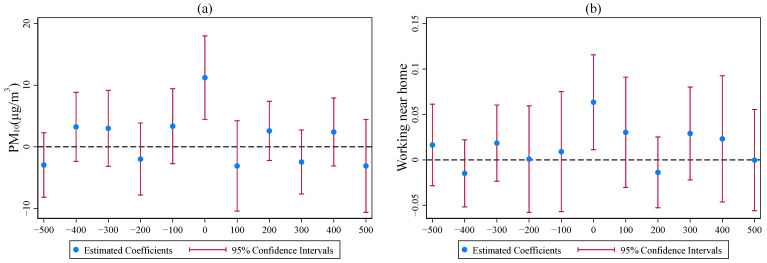
Placebo testing: estimated discontinuity in pollution at displaced Huai River boundaries. Notes: In (**a**), each point plots the point estimate of a separate estimation of *α*_1_ in Equation (2) along with the 95 percent confidence interval at the displaced Huai River boundaries. In (**b**), each point plots the point estimate of a separate estimation of *δ*_1_ in Equation (3) along with the 95 percent confidence interval at the displaced Huai River boundaries. Each RD estimates is based on the MSE bandwidth selection method and triangular kernel function.

Third, we conducted a placebo test to assess the significance of these findings, exploring whether discontinuities are observed in other regions of China. We estimated the discontinuities in PM_10_ and working near home at 100 km intervals north and south of the Huai River across China as well as at the actual Huai River (which is reported as the 0 km displacement). Figure A3 presents estimates and shows that the only statistically significant discontinuous changes in PM_10_ and working near home occur at the actual Huai River. In all other instances, the estimated effect of zero is within the 95% confidence interval. 

**Figure A4:**
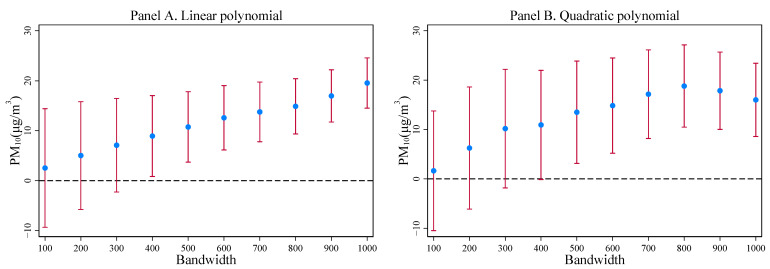
Robustness to alternative bandwidths for the impact of the Huai River Policy on PM_10_. Notes: Each point plots the point estimate of a separate estimation of *α*_1_ in Equation (2) along with the 95 percent confidence interval, ranging from 100 km to 1000 km bandwidths. (**Panel A**) plots estimates using linear polynomials in distance. (**Panel B**) plots estimates from equivalent regressions but using second-order polynomials in distance. Each RD estimate is based on the MSE bandwidth selection method and triangular kernel function.

**Figure A5:**
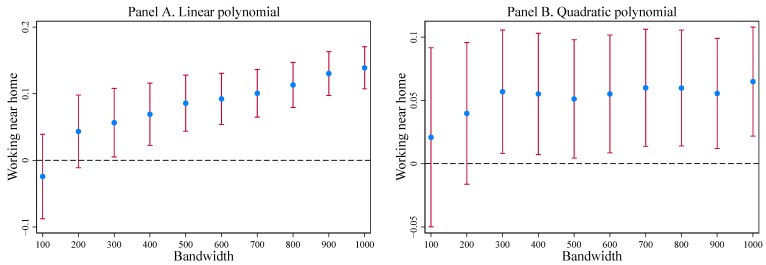
Robustness to alternative bandwidths for the impact of the Huai River Policy on work. Notes: Each point plots the point estimate of a separate estimation of *δ*_1_ in Equation (3) along with the 95 percent confidence interval, ranging from 100 km to 1000 km bandwidths. (**Panel A**) plots estimates using linear polynomials in distance. (**Panel B**) plots estimates from equivalent regressions but using second-order polynomials in distance. Each RD estimate is based on the MSE bandwidth selection method and triangular kernel function.

**Figure A6:**
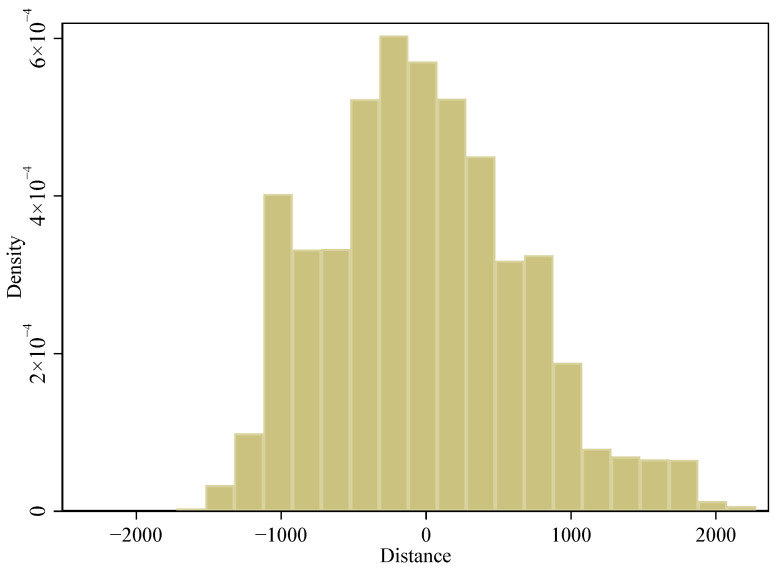
Distributions of distances of counties from the Huai River.

**Figure A7:**
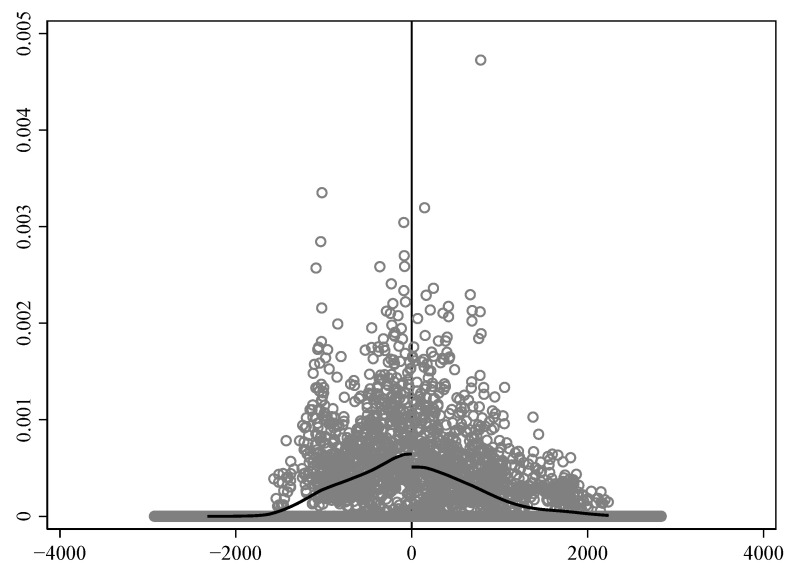
McCrary test of the discontinuity in distances of counties from the Huai River. Notes: The hollow circles represent the point estimates within bins. Solid lines are the density curve estimates.

Fourth, we then examined the sensitivity of our RD estimates to small changes in bandwidths. We set the bandwidths to range from 100 km to 1000 km. For each bandwidth, we estimated the discontinuities in PM_10_ and working near home by local linear regression and second-order polynomial regression, respectively. As shown in Figure A4 and Figure A5 in the Appendix A, the estimate results remain reasonably robust to alternative bandwidths.

Finally, given that air pollution may be responsible for migration in China [15], we explored the potential impact of migration on the results in two ways. One way was to test whether air pollution causes respondents to migrate. We defined migration equal to one if the respondent migrated in the past two years, and zero otherwise, and then investigated the impact of the Huai River Policy and PM_10_ on migration using an RD design. The RD estimates are shown in Table A4. The results in Panel A show that there is no difference in mobility between the north and the south. The results in Panel B show that air pollution has no effect on population migration. This result is consistent with the results of Ebenstein et al. [12]. They assessed migration patterns in China and found that migration did not appreciably alter people’s lifetime exposure to air pollution. There is little evidence that there are many environmental migrations in China. The second way was to exclude those samples with a residence duration of less than 5 years at the same prefecture city level and re-estimate the models (following Ding et al. [52]). Appendix A Table A5 reports the results of the RD estimates. In general, we found that the results are similar in sign and magnitude to those in Table 2 and Table 3. 

## 6. Heterogeneity Analysis 

To better understand the effect of air pollution on working place choice, we examined different subgroups based on respondents’ demographic characteristics. It is helpful for researchers to further study the research topic and for policy makers to design appropriate policies. 

### 6.1. Gender Difference

We examine the gender difference in Table 4. We compared males and females and found that exposure to PM_10_ had a greater positive effect on females working near home than males in general. Panel A summarize the RD estimates of the Huai River Policy on working near home for both men and women. We estimated that the increase in the probability of working near home at the Huai River boundary is around 5.1% and 6.2% (statistically significant) for males and females, respectively. Panel B summarizes the fuzzy RD estimates of PM_10_ on the probability of respondents working near home for both men and women. A 10-unit increase in PM_10_ will significantly increase the probability of working near home for males and females by 9.8% and 11.1%, respectively. These results indicate that females suffer from air pollution more than males. This is consistent with existing studies which show that women have a higher risk for cognitive and health declines associated with increased exposure to air pollution (e.g., Kim et al. [53]; Zhang et al. [7]; Ding et al. [52]).

### 6.2. Age Group Difference

Second, in Table 5, we investigate the impact of air pollution on working place choice for different age groups. We divided the sample into two age groups: young people (aged < 50 years) and old people (aged ≥ 50 years). Panel A summarizes the RD estimates of the Huai River Policy on working near home for both elderly and young people. We found that the Huai River Policy has a positive and statistically significant impact on working near home for the elderly. The increase in the probability of the elderly working near home at the threshold is around 6.8%. In contrast, the magnitude of the estimates is much smaller and statistically insignificant for the young group. Based on the fuzzy RD results in Panel B, a 10-unit increase in PM_10_ will increase the probability of the elderly working near home by 14.8%. Since the elderly suffer from air pollution resulting from the Huai River Policy [43], they are more sensitive to air pollution and are more likely to choose to work near home to mitigate the negative impact of air pollution on health.

### 6.3. Rural–Urban Difference

We also examined how air pollution affects workplace choice behavior between rural and urban individuals. Samples were divided into two groups by the type of hukou (i.e., household registration): urban group and rural group. Table 6 reports the RD estimates for each group. In Panel A, we find that the Huai River Policy significantly increases the probability of working near home by 18.7% for urban respondents, but insignificantly for rural respondents. The fuzzy RD results in Panel B show that a 10-unit increase in PM_10_ will increase the probability of urban respondent working near home by 25.1%. There are three reasons. First, air pollution in urban areas is more serious than in rural areas [43], because there are more pollution emissions (e.g., industrial emissions) and higher implementation intensities for the Huai River Policy in urban areas. Second, air pollution information is readily available in urban areas, but the same information is difficult to obtain in rural areas. Air pollution information is a key determinant of pollution avoidance and associated health impacts [24,43]. Third, due to the nature of the work and transportation cost, the work of rural individuals is relatively fixed (e.g., work on the field). In urban areas, the traffic is relatively perfect and individuals have more job choices. Thus, the effect of air pollution on working near home for the urban group is more significant than that for the rural group.

### 6.4. Occupation Difference

Last, we examined the occupation difference. Samples were divided into three groups by the type of occupation of respondents: primary industry, secondary industry, and tertiary industry. Table 7 reports the RD estimates for each group. Panel A summarizes the RD estimates of the Huai River Policy for the three groups. We found that the Huai River Policy has a negative and statistically significant impact on working near home for those respondents who work in secondary and tertiary industries. Specifically, the increase in the probability of working near home at the threshold is around 16.1% and 14.7% for those respondents who work in secondary and tertiary industries, respectively. Correspondingly, a 10-unit increase in PM_10_ will increase the probability of working near home by 23.5% and 16.9%, respectively. However, this effect does not exist for those respondents who work in primary industries. This is due to the nature of the primary industries. Primary industries depend on natural conditions (e.g., land and trees), which are immovable. Thus, for those respondents who work in the primary industries, work and workplace cannot be changed at will.

## 7. Discussion

Our RD analysis showed that an additional 10 μg/m^3^ in PM_10_ significantly increases the probability of working near home by 13.6 percent. This implies that, facing high levels of pollution, individuals would choose to work nearby to reduce pollution exposure given that migration is restricted. As far as its mechanism and theoretical framework is concerned, the main explanation for our findings may be that air pollution has a significant negative impact on physical and mental health, and this impact is well known [8,12,17]. This mechanism is supported by many related literature reports. Using the same identification strategy as this article, Chen et al. [2] and Ebenstein et al. [12] found that the winter heating policy raised PM_10_ levels by 46 percent in the region north of the Huai River between 2004 and 2012, causing a reduction in life expectancy of 3.4 years. Individuals can take preventive measures to reduce exposure and mitigate the negative impact of air pollution. Based on sales data on air purifiers, Ito and Zhang [14] estimated that a household is willing to pay $13.40 annually to remove 10 mg/m^3^ of PM_10_ and $32.70 annually to eliminate the increased pollution caused by China’s winter heating policy. Willingness to pay for air quality is one of the risk aversion behaviors. Our findings are consistent with the above literature. To mitigate this negative impact of outdoor air pollution, individuals choose to work near home. If one works near home, which means that they are less likely to drive to work and need a shorter amount of time to get to work, they can be less exposed to air pollution. This is a natural and instinctive response to air pollution. Our findings confirm and expand the conclusions of the existing literature. After 2013, China’s real-time pollution monitoring and disclosure program (henceforth, the information program) was launched and marked a turning point in pollution information access and awareness [24]. Therefore, individuals can more easily obtain air quality information and pay attention to health. Based on personal welfare and utility maximization, individuals are more likely to choose to work near home to reduce pollution exposure.

On the other hand, in terms of workplace choice behavior, our results show that in areas with more serious pollution, the labor force tends to work in local cities rather than across regions. A natural question is, what does that mean? A direct result and interpretation is that outdoor air pollution reduces the cross-regional flow of labor and reduces the possibility of labor working across regions. In areas with more serious pollution, the labor mobility is lower, which is an important reason for labor spatial mismatch and market segmentation, as well as further widening of the income gap and unbalanced development among regions [20,40]. In fact, air pollution is aggravating the segmentation of the labor market as a new natural factor [54]. This is a clue that has not been fully studied in the previous literature. The existing studies mainly believe that rivers and terrain are the natural determinants of labor market segmentation [55,56]. In other words, our results show that if air pollution is controlled and reduced, individuals can choose to work further away. This can promote labor mobility and balanced development. These are the theoretical and practical implications of the paper. Our theoretical implication is to build a bridge between environmental economics and labor economics from the perspective of labor mobility. The practical implication is that we have emphasized the necessity of pollution control and that environmental regulation policies should be implemented for a long time.

## 8. Conclusions

Air pollution is considered as a major issue for the community in China. Understanding how changes in pollution levels affect public health and avoidance behaviors is crucial for optimal environmental policy design. We used China’s Huai River Policy as an RD design to evaluate the causal impact of air pollution on working place choice. The Huai River Policy led to the construction of a coal-powered centralized heating infrastructure only in cities north of the Huai River, with no equivalent system in cities to the south. The discontinuity in air pollution caused by the Huai River Policy provides a natural experiment to estimate the impact of air pollution. The data link the 2015 census microdata at the individual level with air pollution at the county level. 

Our results show that the Huai River Policy has increased PM_10_ concentrations by 13.2 percent and caused a 5.6 percent increase in the probability of individuals working near home. This implies that an additional 10 μg/m^3^ in PM_10_ would significantly increase the probability of working near home by 13.6 percent in China, which means that individuals would choose to work nearby to reduce pollution exposure and mitigate the negative impact of pollution on health. Heterogeneity analyses showed that the positive effect of air pollution on the choice to work nearby is more significant for women, the elderly, urban respondents and those respondents who work in secondary and tertiary industries. Following the rich literature, we provided several explanations for our results and discussed the negative impact of air pollution on labor mobility and mismatch by making individuals work nearby. 

The results provide new evidence on how people protect themselves against pollution. Individuals facing high levels of pollution would choose to work nearby to reduce exposure and mitigate the impact. This paper deepens our understanding of coping strategies and avoidance behaviors to environmental shocks and highlights a negative impact on labor mobility and regional balanced development. This is crucial for the regional balanced development policy and environmental policy design in many developing countries. Our results go beyond the trade-off between the economy and environment, and show that worsening air pollution could reduce the potential for economic growth. One policy implication is that to improve the economic quality, air pollution must be further controlled. If air pollution decreases significantly, the resulting rise in labor mobility will promote productivity and achieve a win-win situation for the economy and the environment. One limitation of this paper is that, in recent years, China’s air quality has been continuously improved and the household registration system has been relaxed, and thus people’s risk aversion behavior may change (e.g., migration), which may make the estimation of this paper a higher bound. In future research, we will use updated data and clearer identification strategies to verify the above problems.

## Figures and Tables

**Table 1:** Summary statistics, means, and standard deviations/errors.

Variable	South	North	Difference in Means	Adjusted Difference
	(1)	(2)	(3)	(4)
*Panel A: Air pollution exposure at survey counties*				
PM10	82.748	118.072	35.324 ***	11.225 ***
	(14.116)	(25.527)	(0.044)	(3.459)
*Panel B: Working place selection of respondents*				
Working near home	0.646	0.758	0.112 ***	0.053 **
	(0.478)	(0.428)	(0.001)	(0.027)
*Panel C: Individual characteristics of respondents*				
Gender	0.489	0.488	−0.002	−0.016
	(0.500)	(0.500)	(0.001)	(0.024)
Nation	0.057	0.021	−0.036 ***	−0.015
	(0.232)	(0.144)	(0.000)	(0.010)
Age	38.870	36.790	−2.080 ***	−2.281
	(20.570)	(20.799)	(0.045)	(1.599)
Marriage	0.742	0.750	0.008 ***	−0.007
	(0.438)	(0.433)	(0.001)	(0.010)
Hukou	0.435	0.308	−0.127 ***	−0.246
	(0.496)	(0.462)	(0.001)	(0.146)
*Panel D: Meteorological conditions at survey counties*				
Temperature	16.510	13.569	−2.940 ***	0.094
	(1.201)	(1.653)	(0.003)	(0.154)
Precipitation	9.498	8.879	−0.619 ***	−0.205
	(0.290)	(0.182)	(0.001)	(0.131)
Relative humidity	77.127	65.521	−11.607 ***	0.776
	(2.916)	(5.033)	(0.009)	(0.657)
Wind speed	1.862	2.225	0.362 ***	−0.291
	(0.489)	(0.462)	(0.001)	(0.273)

Note: SDs for means and standard errors for mean differences are in parentheses. Adjusted differences in column (4) are the estimated discontinuity along the Huai River using local linear regression discontinuity with a triangular kernel and the MSE bandwidth selection method. ** Significant at 5%; *** significant at 1%.

**Table 2:** RD estimates of the impacts of the Huai River Policy.

Variables	RD Estimates					
	(1)	(2)	(3)	(4)	(5)	(6)
*Panel A: Impact of the Huai River Policy on PM10*						
PM10	11.225 ***	12.137 ***	12.213 ***	11.784 ***	12.603 ***	12.258 ***
	(3.459)	(3.447)	(4.750)	(3.367)	(3.360)	(4.706)
Bandwidth	538.312	500.452	455.471	546.285	507.273	452.124
*Panel B: Impact of the Huai River Policy on working near home*						
Working near home	0.053 **	0.058 **	0.064 **	0.056 **	0.059 **	0.059 **
	(0.027)	(0.028)	(0.030)	(0.026)	(0.028)	(0.029)
Bandwidth	274.914	241.226	208.783	279.352	250.149	218.813
Observations	802,178	802,178	802,178	802,178	802,178	802,178
Controls	N	N	N	Y	Y	Y
Kernel	Triangular	Epanech.	Uniform	Triangular	Epanech.	Uniform

Note: Each cell in the table represents a separate RD estimate along the Huai River using local linear regressions with different kernel functions. Robust standard errors in parentheses are clustered at the county level. Controls include weather information and sociodemographic variables defined in Table 1. ** Significant at 5%; *** significant at 1%.

**Table 3:** Fuzzy RD and OLS estimates of the impacts of PM_10_ on work.

Variables	Panel A: Fuzzy RD Estimates						Panel B: OLS Estimates	
	(1)	(2)	(3)	(4)	(5)	(6)	(1)	(2)
PM10 (per 10 points)	0.132 ***	0.157 ***	0.152 **	0.136 ***	0.155 ***	0.151 **	0.005 ***	0.005 ***
	(0.043)	(0.046)	(0.068)	(0.043)	(0.042)	(0.064)	(0.001)	(0.001)
Bandwidth	401.273	396.563	374.286	412.204	415.305	345.638		
Observations	802,178	802,178	802,178	802,178	802,178	802,178	802,178	802,178
Controls	N	N	N	Y	Y	Y	N	Y
Kernel	Triangular	Epanech.	Uniform	Triangular	Epanech.	Uniform		

Note: Each cell in the table represents a separate estimate or regression. Columns (1)–(6) report the fuzzy RD results estimating the impact of 10 μg/m^3^ of PM_10_ on work, treating distance from the Huai River as the forcing variable and PM_10_ as the treating variable, with the Huai River representing a “fuzzy” discontinuity in the level of air pollution exposure. Column (1) and (2) in Panel B report the OLS estimates of the association between PM_10_ and work. Robust standard errors in parentheses are clustered at the county level. Controls include weather information and sociodemographic variables defined in Table 1. ** Significant at 5%; *** significant at 1%.

**Table 4:** The impacts of PM_10_ on work by gender.

Variables	Panel A: Impacts of Huai River Policy on Work				Panel B: Impacts of PM10 on Work			
	Males		Females		Males		Females	
	(1)	(2)	(3)	(4)	(1)	(2)	(3)	(4)
Working near home	0.051 **	0.045 *	0.062 **	0.087 ***	0.098 *	0.079 *	0.111 **	0.090 *
	(0.026)	(0.024)	(0.029)	(0.033)	(0.051)	(0.043)	(0.054)	(0.052)
Bandwidth	311.742	264.165	278.618	297.873	307.526	229.815	291.463	233.927
Observations	463,585	463,585	338,593	338,593	463,585	463,585	338,593	338,593
Controls	Y	Y	Y	Y	Y	Y	Y	Y
Kernel	Triangular	Uniform	Triangular	Uniform	Triangular	Uniform	Triangular	Uniform

Note: Each cell in the table represents a separate estimate or regression. Panel A reports the RD results estimating the impact of the Huai River Policy on work. Panel B reports the fuzzy RD results estimating the impact of 10 μg/m^3^ of PM_10_ on work, treating distance from the Huai River as the forcing variable and PM_10_ as the treating variable, with the Huai River representing a “fuzzy” discontinuity in the level of air pollution exposure. Robust standard errors in parentheses are clustered at the county level. Controls include weather information and sociodemographic variables defined in Table 1. * Significant at 10%; ** significant at 5%; *** significant at 1%.

**Table 5:** The impacts of PM_10_ on work by age.

Variables	Panel A: Impacts of Huai River Policy				Panel B: Impacts of PM10 on Work			
	Age < 50		Age ≥ 50		Age < 50		Age ≥ 50	
	(1)	(2)	(3)	(4)	(1)	(2)	(3)	(4)
Working near home	0.048	0.049	0.068 ***	0.093 ***	0.100	0.115	0.148 **	0.144 **
	(0.030)	(0.033)	(0.020)	(0.020)	(0.133)	(0.327)	(0.074)	(0.065)
Bandwidth	291.291	206.563	241.578	319.535	279.627	167.415	284.219	314.158
Observations	600,252	600,252	201,926	201,926	600,252	600,252	201,926	201,926
Controls	Y	Y	Y	Y	Y	Y	Y	Y
Kernel	Triangular	Uniform	Triangular	Uniform	Triangular	Uniform	Triangular	Uniform

Note: Each cell in the table represents a separate estimate or regression. Panel A reports the RD results estimating the impact of the Huai River Policy on work. Panel B reports the fuzzy RD results estimating the impact of 10 μg/m^3^ of PM_10_ on work, treating distance from the Huai River as the forcing variable and PM_10_ as the treating variable, with the Huai River representing a “fuzzy” discontinuity at the level of air pollution exposure. Robust standard errors in parentheses are clustered to the county level. Controls include weather information and sociodemographic variables defined in Table 1. ** Significant at 5%; *** significant at 1%.

**Table 6:** The impacts of PM_10_ on work by rural–urban.

Variables	Panel A: Impacts of Huai River Policy				Panel B: Impacts of PM10 on Work			
	Rural		Urban		Rural		Urban	
	(1)	(2)	(3)	(4)	(1)	(2)	(3)	(4)
Working near home	0.025	0.027	0.187 ***	0.197 ***	0.045	0.034	0.251 **	0.185 *
	(0.025)	(0.025)	(0.043)	(0.046)	(0.055)	(0.048)	(0.128)	(0.098)
Bandwidth	293.338	238.894	310.852	251.616	276.526	204.354	285.683	352.119
Observations	554,756	554,756	247,422	247,422	554,756	554,756	247,422	247,422
Controls	Y	Y	Y	Y	Y	Y	Y	Y
Kernel	Triangular	Uniform	Triangular	Uniform	Triangular	Uniform	Triangular	Uniform

Note: Each cell in the table represents a separate estimate or regression. Panel A reports the RD results estimating the impact of the Huai River Policy on work. Panel B reports the fuzzy RD results estimating the impact of 10 μg/m^3^ of PM_10_ on work, treating distance from the Huai River as the forcing variable and PM_10_ as the treating variable, with the Huai River representing a “fuzzy” discontinuity in the level of air pollution exposure. Robust standard errors in parentheses are clustered at the county level. Controls include weather information and sociodemographic variables defined in Table 1. * Significant at 10%; ** significant at 5%; *** significant at 1%.

**Table 7:** The impacts of PM_10_ on work by occupation.

Variables	Panel A: Impacts of Huai River Policy			Panel B: Impacts of PM10 on Work		
	Ind1	Ind2	Ind3	Ind1	Ind2	Ind3
	(1)	(2)	(3)	(1)	(2)	(3)
Working near home	0.003	0.161 ***	0.147 ***	0.001	0.235 ***	0.169 ***
	(0.004)	(0.040)	(0.030)	(0.004)	(0.067)	(0.055)
Bandwidth	399.085	342.017	379.282	315.258	349.973	373.902
Observations	263,564	235,168	303,446	263,564	235,168	303,446
Controls	Y	Y	Y	Y	Y	Y
Kernel	Triangular	Triangular	Triangular	Triangular	Triangular	Triangular

*Note*: Each cell in the table represents a separate estimate or regression. Panel A reports the RD results estimating the impact of the Huai River Policy on work. Panel B reports the fuzzy RD results estimating the impact of 10 μg/m^3^ of PM_10_ on work, treating distance from the Huai River as the forcing variable and PM_10_ as the treating variable, with the Huai River representing a “fuzzy” discontinuity in the level of air pollution exposure. Robust standard errors in parentheses are clustered at the county level. Controls include weather information and sociodemographic variables defined in Table 1. *** Significant at 1%.

## Data Availability

3rd Party Data. Restrictions apply to the availability of these data. Data was obtained from National Bureau of Statistics in China and are available with the permission of School of Economics, Shanghai University of Finance and Economics.

## Appendix A

**Table A1:** Descriptive statistics.

Variables	Obs	Mean	Std. Dev	Min	Max
*Working place selection*					
Working near home (Yes = 1)	802,178	0.702	0.458	0.000	1.000
*Air pollution*					
PM_10_ concentration (μg/m^3^)	802,178	89.241	30.496	25.192	181.676
*Individual characteristics*					
Gender (Male = 1)	802,178	0.487	0.500	0.000	1.000
Nation (Han = 1)	802,178	0.086	0.281	0.000	1.000
Age	802,178	37.364	20.593	0.000	111.000
Marriage (Yes = 1)	802,178	0.735	0.441	0.000	1.000
Type of hukou (Urban = 1)	802,178	0.410	0.492	0.000	1.000
*Meteorological conditions*					
Temperature of county (°C)	802,178	3.627	3.883	24.473	3.627
Precipitation of county (mm)	802,178	0.508	0.000	10.118	0.508
Relative humidity	802,178	9.008	34.465	86.018	9.008
Wind speed (m/s)	802,178	0.515	0.931	5.845	0.515

Note: This table reports the summary statistics for the main variables used in the analysis.

**Table A2:** Robustness to using the alternative air pollution measure (AQI).

Variables	RD Estimates					
	(1)	(2)	(3)	(4)	(5)	(6)
*Panel A: Impact of the Huai River Policy on AQI*						
AQI	13.241 ***	12.781 ***	12.238 ***	13.331 ***	11.942 ***	12.852 ***
	(3.032)	(3.103)	(4.328)	(4.219)	(3.534)	(3.163)
Bandwidth	494.842	503.716	467.435	473.853	482.471	453.289
*Panel B: Impact of the AQI on working near home*						
Working near home	0.142 **	0.148 **	0.161 **	0.136 **	0.141 **	0.157 **
	(0.061)	(0.065)	(0.067)	(0.063)	(0.064)	(0.062)
Bandwidth	478.537	486.562	469.364	459.817	467.384	439.715
Observations	802,178	802,178	802,178	802,178	802,178	802,178
Controls	N	N	N	Y	Y	Y
Kernel	Triangular	Epanech.	Uniform	Triangular	Epanech.	Uniform

Note: Each cell in the table represents a separate RD estimate along the Huai River using local linear regressions with different kernel functions. Robust standard errors in parentheses are clustered at the county level. Controls include weather information and sociodemographic variables defined in Table 1. ** Significant at 5%; *** significant at 1%.

**Table A3:** RD estimates of the impacts on the alternative work measure.

Variables	Panel A: Impact of the Huai River Policy on Work		Panel B: Impacts of PM10 on Work	
	(1)	(2)	(1)	(2)
Driving to work	−0.128 ***	−0.124 ***	−0.244 **	−0.242 **
	(0.013)	(0.014)	(0.113)	(0.119)
Bandwidth	365.666	316.909	296.336	297.601
Time required	−3.709 ***	−3.781 ***	−7.186 ***	−7.499 **
	(1.263)	(1.251)	(2.195)	(2.927)
Bandwidth	354.039	356.565	233.738	229.445
Observations	802,178	802,178	802,178	802,178
Controls	N	Y	N	Y
Kernel	Triangular	Triangular	Triangular	Triangular

Note: Each cell in the table represents a separate estimate or regression. Panel A reports the RD results estimating the impact of the Huai River Policy on work. Panel B reports the fuzzy RD results estimating the impact of 10 µg/m^3^ of PM_10_ on work, treating distance from the Huai River as the forcing variable and PM_10_ as the treating variable, with the Huai River representing a “fuzzy” discontinuity in the level of air pollution exposure. The triangular kernel function is used. Robust standard errors in parentheses are clustered at the county level. Controls include weather information and sociodemographic variables defined in Table 1. ** Significant at 5%; *** significant at 1%.

**Table A4:** RD estimates of the impacts on migration.

Variables	Panel A: Impact of the Huai River Policy on Migration		Panel B: Impacts of PM10 on Migration	
	(1)	(2)	(1)	(2)
Migration	0.053	0.070	0.146	0.195
	(0.122)	(0.123)	(0.216)	(0.271)
Bandwidth	219.60	243.68	218.91	228.66
Observations	802,178	802,178	802,178	802,178
Controls	N	Y	N	Y
Kernel	Triangular	Triangular	Triangular	Triangular

Note: Each cell in the table represents a separate estimate or regression. Panel A reports the RD results estimating the impact of the Huai River Policy on migration. Panel B reports the fuzzy RD results estimating the impact of 10 µg/m^3^ of PM_10_ on migration, treating distance from the Huai River as the forcing variable and PM_10_ as the treating variable, with the Huai River representing a “fuzzy” discontinuity in the level of air pollution exposure. The triangular kernel function is used. Robust standard errors in parentheses are clustered at the county level. Controls include weather information and sociodemographic variables defined in Table 1.

**Table A5:** RD estimates for samples with a residence duration at the same prefecture-level city of more than 5 years.

Variables	Panel A: Impact of the Huai River Policy on Work		Panel B: Impacts of PM10 on Work	
	(1)	(2)	(1)	(2)
Working near home	0.051 **	0.054 **	0.128 ***	0.124 ***
	(0.022)	(0.027)	(0.041)	(0.042)
Bandwidth	277.742	279.925	431.48	427.57
Observations	698,504	698,504	698,504	698,504
Controls	N	Y	N	Y
Kernel	Triangular	Triangular	Triangular	Triangular

Note: Each cell in the table represents a separate estimate or regression. Panel A reports the RD results estimating the impact of the Huai River Policy on work. Panel B reports the fuzzy RD results estimating the impact of 10 µg/m^3^ of PM_10_ on work, treating distance from the Huai River as the forcing variable and PM_10_ as the treating variable, with the Huai River representing a “fuzzy” discontinuity in the level of air pollution exposure. The triangular kernel function is used. Robust standard errors in parentheses are clustered at the county level. Controls include weather information and sociodemographic variables defined in Table 1. ** significant at 5%; *** significant at 1%.
